# Anti-SARS-CoV-2 glyco-humanized polyclonal antibody XAV-19: phase II/III randomized placebo-controlled trial shows acceleration to recovery for mild to moderate patients with COVID-19

**DOI:** 10.3389/fimmu.2024.1330178

**Published:** 2024-04-17

**Authors:** Garyfallia Poulakou, Pierre-Joseph Royer, Nikolay Evgeniev, Gwénaëlle Evanno, Françoise Shneiker, Anne-Geneviève Marcelin, Bernard Vanhove, Odile Duvaux, Stéphane Marot, Vincent Calvez

**Affiliations:** ^1^ 3rd Department of Internal Medicine, Medical School, Sotiria General Hospital, National and Kapodistrian University of Athens, Athens, Greece; ^2^ Xenothera, Nantes, France; ^3^ Department of Medical Oncology, Complex Oncology Center, Russe, Bulgaria; ^4^ Sorbonne Université, Institut National de la Santé et de la Recherche Médicale (INSERM) 1136, Institut Pierre Louis d’Epidémiologie et de Santé Publique (iPLESP), Assistance Publique-Hôpitaux de Paris (AP-HP), Pitié Salpêtrière Hospital, Department of Virology, Paris, France

**Keywords:** COVID-19, clinical trial, (glyco-humanized) polyclonal antibody, XAV-19, SARS-CoV-2 variants

## Abstract

**Introduction:**

XAV-19 is a glyco-humanized swine polyclonal antibody targeting SARS-CoV-2 with high neutralizing activity. The safety and clinical efficacy of XAV-19 were investigated in patients with mild to moderate COVID-19.

**Methods:**

This phase II/III, multicentric, randomized, double-blind, placebo-controlled clinical trial was conducted to evaluate the safety and clinical efficacy of XAV-19 in patients with a seven-point WHO score of 2 to 4 at randomization, i.e., inpatients with COVID-19 requiring or not requiring low-flow oxygen therapy, and outpatients not requiring oxygen (EUROXAV trial, NCT04928430). Adult patients presenting in specialized or emergency units with confirmed COVID-19 and giving their consent to participate in the study were randomized to receive 150 mg of XAV-19 or placebo. The primary endpoint was the proportion of patients with aggravation within 8 days after treatment, defined as a worsening of the seven-point WHO score of at least one point between day 8 and day 1 (inclusion). The neutralization activity of XAV-19 against variants circulating during the trial was tested in parallel.

**Results:**

From March 2021 to October 2022, 279 patients received either XAV-19 (N = 140) or placebo (N = 139). A slow enrollment and a low rate of events forced the termination of the premature trial. XAV-19 was well tolerated. Underpowered statistics did not allow the detection of any difference in the primary endpoint between the two groups or in stratified groups. Interestingly, analysis of the time to improvement (secondary endpoint) showed that XAV-19 significantly accelerated the recovery for patients with a WHO score of 2 or 3 (median at 7 days vs. 14 days, p = 0.0159), and even more for patients with a WHO score of 2 (4 days vs. 14 days, p = 0.0003). The neutralizing activity against Omicron and BA.2, BA.2.12.1, BA.4/5, and BQ.1.1 subvariants was shown.

**Discussion:**

In this randomized placebo- controlled trial with premature termination, reduction of aggravation by XAV-19 at day 8 in patients with COVID-19 was not detectable. However, a significant reduction of the time to improvement for patients not requiring oxygen was observed. XAV-19 maintained a neutralizing activity against SARS-CoV-2 variants. Altogether, these data support a possible therapeutic interest for patients with mild to moderate COVID-19 requiring anti-SARS-CoV-2 neutralizing antibodies.

**Clinical Trial Registration:**

https://clinicaltrials.gov/, identifier NCT04928430; https://www.clinicaltrialsregister.eu/about.html (EudraCT), identifier 2020-005979-12.

## Highlights

- In this phase II/III randomized placebo-controlled clinical trial including 279 patients hospitalized or not, with mild to moderate COVID-19 (seven-point ordinal WHO score of 2, 3, or 4 at randomization), treatment with XAV-19 significantly accelerated recovery defined by a reduction of one point or more based on the seven-point ordinal scale.
- The strengths of XAV-19 are its affordability and its activity against SARS-CoV-2 variants.
- Succeeding trials should confirm the therapeutic interest of XAV-19 for high-risk patients with mild to moderate COVID-19.

## Introduction

Since the beginning of the coronavirus disease 2019 (COVID-19) pandemic, caused by severe acute respiratory syndrome coronavirus-2 (SARS-CoV-2), mass vaccination campaigns have dramatically reduced the impact of the pandemic and the number of deaths. Prophylactic or therapeutic drugs such as antibodies, small molecules, or natural dietary compounds are essential complements to vaccines (1–4). They are particularly needed for immunocompromised patients or people who do not respond to the vaccination (5, 6).

Numerous neutralizing monoclonal antibodies (mAbs) have been developed against SARS-CoV-2. Most of them were raised against the original Wuhan-type virus and see their neutralization potential abrogated or reduced against variants. Moreover, mAbs may favor the emergence of SARS-CoV-2-resistant variants in immunocompromised patients (7). Approved anti-SARS-CoV-2 mAbs are no longer recommended since the end of 2022 because they are unlikely to be effective against emerging strains of SARS-CoV-2 (8–10). Passive immunotherapy with convalescent COVID-19 plasma has also been investigated, although many questions remain regarding the clinical efficiency of this strategy and its large-scale feasibility, i.e., donor selection, batch-to-batch reproducibility, or safety issues (11–13). Owing to their potential to bind multiple target epitopes and maintain their neutralizing activity despite mutations, polyclonal antibodies (pAbs) of animal origin represent a promising approach against COVID-19 (14, 15). Batch-to-batch reproducibility and viral safety are critical points in pAb production. Hyperimmunization of qualified and selected animals guarantees large volumes of high-titer and controlled pAb (15), and the purification process ensures less than one viral particle per 6 million doses (16). Thereby, pAbs against SARS-CoV-2 are being tested in clinical trials (13, 17, 18).

XAV-19 is a swine glyco-humanized polyclonal antibody (GH-pAb) issued from our technological platform as previously described elsewhere (15, 16). XAV-19 is directed against the Wuhan-type SARS-CoV-2 receptor-binding domain (RBD). Thanks to the removal of xeno-antigens, XAV-19 is likely to prevent post-infusion serum sickness and allergies (19, 20).

XAV-19 broadly neutralizes variants and avoids selection of variants (15); it has been introduced in clinics since 2020. No hypersensitivity or infusion-related reactions were reported during treatment, and there were no discontinuations for adverse events (AEs) and no serious adverse events (SAEs) related to the study drug in a phase IIa clinical trial for inpatients under oxygen (21). Here, we investigated the clinical impact of XAV-19 for patients (inpatients or outpatients) suffering from mild to moderate COVID-19. The results of the EUROXAV study (NCT04928430, EudraCT: 2020-005979-12), an international, placebo-controlled, double-blind, randomized clinical trial designed to evaluate the efficacy and safety of XAV-19 in patients with moderate COVID-19 requiring or not requiring oxygen therapy, are presented. Moreover, as SARS-CoV-2 continues to evolve, the activity of XAV-19 against subvariants spreading during the trial was tested *in vitro*.

## Methods

### Phase II/III trial

#### Study design

EUROXAV (NCT04928430; EudraCT: 2020-005979-12) was a multicenter, international phase II/III, double-blind (patient and clinician), placebo-controlled, randomized clinical trial conducted in 14 hospitals from five countries (Bulgaria, Greece, Romania, Spain, and Turkey) between March 2021 and October 2022. This trial followed the International Council for Harmonization E6 guideline for good clinical practice and the principles of the Declaration of Helsinki. An independent Data and Safety Monitoring Board (DSMB) examined the data after 200 and 400 inclusions and on any demand from the sponsor.

#### Participants

Adult patients having SARS-CoV-2-confirmed infection (positive RT-PCR, RT-qPCR, or antigen test in the last 10 days) and presenting signs of respiratory disease with at least two clinical symptoms related to COVID-19 (fever, cough, sore throat, nasal discharge, dyspnea, thoracic pain, headache or fatigue, myalgia, anosmia, dysgeusia, diarrhea, and nausea) that started less than 10 days prior to screening visit were eligible. Patients should have SpO_2_ >90% at ambient air and require or not low-flow oxygen therapy [a score of 2, 3, or 4 on the WHO clinical progression seven-point ordinal scale: 1, not hospitalized, no limitations on activities; 2: not hospitalized, limitations on activities; 3: hospitalized, not requiring supplemental oxygen; 4: hospitalized, requiring supplemental oxygen; 5: hospitalized, on non-invasive ventilation (NIV) or high-flow oxygen device; 6: hospitalized, on invasive mechanical ventilation or extracorporeal membrane oxygenation (ECMO); 7: death]. Exclusion criteria were as follows: positive SARS-CoV-2 test >10 days, multiorgan failure, immediate intensive care unit (ICU) hospitalization, critical respiratory illness (high-flow oxygenation, NIV, invasive mechanical ventilation, or ECMO), requirement of oxygenation at a flow rate of >6 L/min, signs of severe systemic illness (respiratory rate ≥30/min, heart rate ≥ 125/min, and PaO_2_/FiO_2_ < 300), participation in another trial, pregnancy, or breastfeeding. Patients with prior anti-COVID-19 vaccine were not excluded whatever the delay. Vaccination was not permitted for a patient during the study before 90 days after the acute COVID episode.

#### Randomization and intervention

Eligible patients were randomized in a 1:1 ratio to receive either XAV-19 (150 mg) diluted in sterile NaCl 0.9% or placebo (NaCl 0.9% only) as a 1-h intravenous perfusion. Randomization was stratified by center and by WHO score, the list being established using the SAS^®^ software and allocated by an Interactive Web Response System. Patients were monitored during the infusion and the following hour. Per the investigator’s judgment, the patient was either hospitalized or discharged (ambulatory patients). Each center was allowed to prescribe dexamethasone 6 mg/day orally or intravenously for 10 days or until hospital discharge, antithrombotic prophylaxis [dose adapted to the body mass index (BMI)], antibiotic therapy, and any antiviral medication (except anti-SARS-CoV-2 mAbs or convalescent patients’ plasma) according to the judgment of the investigator and to the national guidelines or best standard of care. Concomitant treatments were collected in the eCRF. Patients were followed up at days 3, 5, 8, 15, and 29. Visits on days 3, 5, and 29 were either on site for inpatients or by phone for outpatients. Visits on days 8 and 15 were on site for all patients.

#### Endpoints

The primary endpoint was the proportion of patients with an aggravation of COVID-19 within 8 days. The aggravation was defined as a worsening of the score of at least one point on the WHO seven-point ordinal scale compared to the score at day 1 (inclusion). Main secondary endpoints were the proportion of patients with an aggravation of two points of COVID-19 within 8 days; the proportion of patients with an aggravation of one or two points within 15 days; time to aggravation; time to improvement, defined as the time of first documentation of aggravation/improvement; length of hospital stay; the proportion of patients transferred to ICU or needing mechanical ventilation; and overall survival. Safety outcomes included the cumulative incidence of all types of AEs. Planned exploratory endpoints were pharmacokinetics (Cmax, T1/2, and AUC), anti-drug antibodies, and SARS-CoV-2 neutralizing antibody titers.

#### Statistical analyses

Sample size calculation was based on the following hypotheses: 15% aggravation of COVID-19 at day 8 in the placebo arm and 8% in the XAV-19 arm; type I error set at 5%, two-sided test, and power set at 80%. Applying a drop-out rate of 25% to consider non-eligible or early withdrawn patients, the number of patients to be recruited was established at 870 (435 per arm). Binary logistic regression model was performed for the primary efficacy analysis. Association between the randomization group and the progression of COVID-19 was adjusted on center, WHO score, country, age, BMI, gender, and comorbidities (defined as BMI >30, diabetes DNID, diabetes DID, cardiac disorder, vascular disorder, hypercholesterolemia, renal failure, lung disease COPD, and/or asthma). The dependent variable was the aggravation of COVID-19 within 8 days after treatment initiation, and the model included the following factors: treatment group, center, WHO score at baseline (2–3 vs. 4), country, age, comorbidities (yes/no), BMI, and gender. PROC LOGISTIC was used to perform logistic regression. The comparisons between the XAV-19 group and the placebo group of two or more qualitative variables were made using the χ^2^ test, the continuity-corrected χ^2^ test, or the Fisher exact test, according to the expected values under the assumption of independence. Comparisons of quantitative variables between the XAV-19 and placebo groups were made using a Student *t*-test (parametric test comparing means) or Mann–Whitney–Wilcoxon test (non-parametric test comparing ranks) depending on the distribution of the variable of interest. A log rank test was used to compare time to aggravation/improvement between randomization groups. Kaplan–Meier curves are presented by randomization groups. Different populations were used in the analysis: a Safety population including all subjects randomized who received the product; an Intent-to-treat (ITT) population including all randomized patients regardless of their eligibility and any protocol deviations; and the Target population, the main one for all efficacy analysis, including ITT patients having received the treatment and fulfilling the main inclusion and non-inclusion criteria, i.e., aged 18 or older, weighing between 40 and 120 kg at the time of signing the informed consent, and requiring or not requiring low-flow oxygen therapy with a WHO score of 2, 3, or 4. For missing data, the strategy “missing = failure” was applied for patients who withdrew before day 8 or for patients with missing data. A sensitivity analysis was performed with the Last Observation Carry Forward (LOCF) hypothesis of replacement of missing data. The baseline (inclusion) value of the WHO score was defined as the last value before randomization. If the value was missing, the value of the screening visit is applied. All statistical analyses were performed with SAS Studio version 5.2.

#### Safety

All AEs, adverse drug reactions (ADRs), SAEs, serious adverse reaction (SARs), and unexpected adverse drug reaction (UADRs) were recorded. Grading was established according to the CTCAE.

### *In vitro* antiviral activity

All methods have been described elsewhere (15).

RBD Binding ELISA: recombinant His-Tag-RBD of the Wuhan type (40592-V08H) or of the Omicron (40592-V08H121), BA.2 (40592-V08H123), BA.2.12.1 (40592-V08H132), BA.4/5 (40592-V08H130), and BQ.1.1 (40592-V08H143) mutants was purchased from Sino Biological (Eschborn, Germany). Recombinant His-Tag-RBD was immobilized on Maxisorp (Nunc) plates. After washes and saturation, successive dilutions of XAV-19 or Evusheld^®^ (tixagevimab-AZD8895/cilgavimab-AZD1060, AstraZeneca, Cambridge, UK) were added. After three washes, porcine or human immunoglobulins were detected with a peroxidase-conjugated anti-pig (MT424, Mabtech, Nacka Strand, Sweden) or anti-human (705-035-147, Jackson, Cambridge, UK) secondary antibody. Binding was revealed by the addition of TMB (Sigma, France). Optical density was read at 450 nm with a TECAN plate reader.

Spike/ACE-2 neutralization assay: briefly, recombinant His-Tag-RBD pre-incubated with XAV-19 or Evusheld^®^ were added to human ACE2-coated plates. Bound RBD was then detected with HRP-conjugated anti-His-Tag antibody and revealed using a peroxidase-conjugated anti-mouse secondary antibody. Binding intensity was revealed by the addition of TMB. Optical density was taken at 450 nm.

Cytopathogenic effect (CPE): briefly, SARS-CoV-2 stocks from clinical isolates were generated by one passage on Vero cells and titered by limiting dilution assay and allowing the calculation of tissue culture infective dose 50% (TCID50). Serial dilutions of XAV-19 were incubated with virus (2 × 10^3^ TCID50/mL) in eight replicates. Vero E6 cells were then added to the mixture and incubated until microscopy examination on day 4 to assess CPE. For viral load quantification, RNA was extracted from the eight pooled replicates of each XAV-19 dilution and the relative viral loads were assessed by quantification of the ORF1ab gene with the TaqPath™ COVID-19 RT-PCR kit (ThermoFisher, Waltham, USA) after linear regression in log10 copies/mL with a standard curve realized from a SARS-CoV-2-positive nasopharyngeal sample quantified by Droplet-Digital PCR (Bio-Rad, Marnes-la-Coquette, France).

## Results

### Demography and patient characteristics

**Figure 1:**
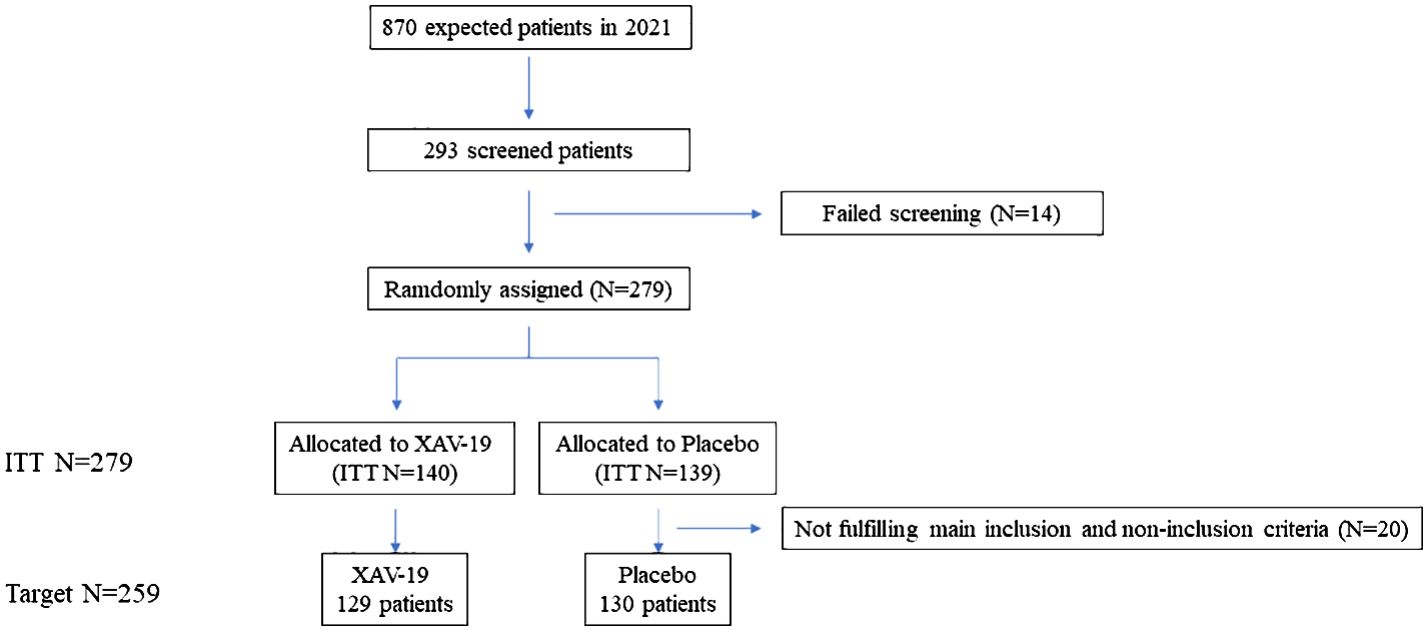
Flow of participants in the EUROXAV phase II/III clinical trial, an international, placebo-controlled, double-blind, randomized clinical trial to evaluate the efficacy and safety of 150 mg of XAV-19 infusion, in patients with mild to moderate COVID-19.

EUROXAV was initiated in March 2021 and expected to enroll 870 patients in 18 centers in Greece, Bulgaria, Romania, Turkey, and Spain. Most patients were enrolled in 2021, and recruitment turned very slow in 2022. The total event rate was also much lower than expected (8% observed vs. 15% expected). A blind interim analysis was shared with the DSMB who recommended to stop the study, which was done. A total of 293 patients with confirmed COVID-19 had been screened; among them, 279 were randomized to receive XAV-19 or placebo (*n* = 139 vs. 140, ITT population). Twenty patients (10 patients in each arm) were not kept for further analysis in the Target population due to lack of treatment or a WHO score of 1 at randomization (**Figure 1**). Patients’ characteristics are presented in **Table 1**. In XAV-19/placebo arms, respectively, median age was 58.0/56.0 years, the percentage of male patients was 57.4%/51.5%, median BMI was 27.7/27.3, the proportion of smokers (past and current) was 27.5%/25.8%, and biological parameters were similar (not shown). Comorbidities were slightly higher in the XAV-19 group (62% vs. 49.2%) and the percentage of immunocompromised patients was 8.5% in both groups. For COVID-19 initial status, both groups were similar for WHO score at day 1: 25.6%/25.4% at a score of 2, 45.7%/46.2% at a score of 3, and 28.7%/28.5% at a score of 4; symptom onset was similar at 5.0/6.0 days, and time between positive test and inclusion was very short (1.0/1.0 days). Blood pressure measures were equivalent (systolic 125.1/124.8 mmHg, diastolic 76.5/75.9 mmHg) and mean body temperature was normal in both groups. As established by chest x-ray, CT scan, or auscultation, 94.6% vs. 93% of patients had signs of pneumonia, and SpO_2_ at ambient air was 94.9% vs. 94.7%, respectively. The trial was initiated before any vaccine was available in the countries participating; thus, the investigator’s estimation is that less than 2% of the patients might have been vaccinated. Concomitant treatments were anticoagulant for 42.6%/39.2% of the patients and glucocorticoid for 27.9%/26.2%, respectively.

**Table 1:** Demographic and disease characteristics by treatment group at day 1 (inclusion).

Characteristic	XAV-19 (*N* = 129)	Placebo (*N* = 130)	Total (*N* = 259)
**Age at screening, median (Q1–Q3)**	58.0 (47.0–70.0)	56.0 (45.0–66.0)	57.9 (46.0–69.0)
**Gender: male patients, *n* (%)**	74 (57.4)	67 (51.5)	141 (54.4)
**BMI, median (Q1–Q3)**	27.7 (25.2–31.3)	27.3 (24.8–31.0)	27.5 (24.9–31.1)
**Current smoker, *n* (%)** **Past smoker *n* (%)**	14 (11.0) 21 (16.5)	16 (12.5) 17 (13.3)	30 (11.8) 38 (14.9)
**Comorbidities, *n* (%)**	80 (62.0)	64 (49.2)	144 (55.6)
**Immunocompromised patients, *n* (%)**	11 (8.5)	11 (8.5)	22 (8.5)
WHO score at day 1, *n* (%)			
WHO 2	33 (25.6)	33 (25.4)	66 (25.5)
WHO 3	59 (45.7)	60 (46.2)	119 (45.9)
WHO 4	37 (28.7)	37 (28.5)	74 (28.6)
**Time between SARS-CoV-2 test and treatment in days, median (Q1–Q3)**	1.0 (1.0–3.0)	1.0 (1.0–4.0)	1.0 (1.0–4.0)
**Onset of oldest COVID-19 symptom in days, median (Q1–Q3)**	5.0 (4.0–7.0)	6.0 (4.0–7.0)	5.0 (4.0–7.0)
Vital signs			
Systolic blood pressure, mmHg, mean (SD)	125.1 (14.6)	124.8 (16.3)	124.9 (15.5)
Diastolic blood pressure, mmHg, mean (SD)	76.5 (9.4)	75.9 (9.8)	76.2 (9.6)
Body temperature, mean (SD)	37.3 (0.7)	37.2 (0.8)	37.3 (0.7)
Respiratory examination			
Signs of pneumonia^¥^, *n* (%)	122 (94.6)	120 (93.0)	242 (93.8)
SpO_2_ at ambient air (%), mean (SD)	94.9 (1.8)	94.7 (2.1)	94.8 (1.9)
Patients under O_2_ supplementation, *n* (%)	37 (28.7)	37 (28.5)	74 (28.6)

^¥^:determined by chest x-ray, CT scan, or auscultation; total differs due to missing values.

### Endpoints

Because of the reduced number of patients, data were expected to deliver limited information. The primary endpoint was the proportion of patients with an aggravation of COVID-19 within 8 days after treatment. No significant difference was detectable between the XAV-19 or placebo groups in the ITT population (worsening representing 8.5% vs. 6.9% in the two arms, respectively) or in the Target population (9.3% vs. 6.9%, with no difference with an LOCF replacement) (not shown). **Table 2** shows bivariate analyses in the Target population. In this trial, gender, BMI, and WHO score were not associated with COVID-19 progression (**Table 2**). In contrast, logistic regression confirmed the association between age over 70 and comorbidities, with aggravation of COVID-19 [age: *p* = 0.001, OR = 6.222 (95% CI: 2.464; 15.712); comorbidities: *p* = 0.0147, OR = 3.715 (95% CI: 1.214; 11.368)].

**Table 2:** Bivariate analysis between categorical variables and aggravation of COVID-19.

Parameters	No worsening	Worsening	Total	*p*-values	OR [95/% CI]
	*N* = 238	*N* = 21	*N* = 259		
Treatment, *n* (%)					
XAV-19	117 (49.2)	12 (57.1)	129 (49.4)		
Placebo	121 (50.8)	9 (42.9)	130 (50.6)	*0.4831*	1.379 [0.560; 3.394]
Age at screening, *n* (%)					
≤50 years	87 (36.6)	2 (9.5)	89 (34.4)		
>50 years	151 (63.4)	19 (90.5)	170 (65.6)	*0.0124*	5.473 [1.245; 24.061]
≤60 years	144 (60.5)	6 (28.6)	150 (57.9)		
>60 years	94 (39.5)	15 (71.4)	109 (42.1)	*0.0045*	3.829 [1.435; 10.221]
≤70 years	196 (82.4)	9 (42.9)	205 (79.2)		
>70 years	42 (17.6)	12 (57.1)	54 (20.8)	*0.0001*	6.222 [2.464; 15.712]
≤80 years	230 (96.6)	18 (85.7)	248 (95.8)		
>80 years	8 (3.4)	3 (14.3)	11 (4.2)	*0.0498*	4.792 [1.169; 19.645]
Gender, *n* (%)					
Male	130 (54.6)	11 (52.4)	141 (54.4)		
Female	108 (45.4)	10 (47.6)	118 (45.6)	*0.8433*	0.914 [0.374; 2.233]
BMI, mean (SD)	28.2 (4.8)	27.8 (2.6)	28.2 (4.7)		
Malnutrition: <18.5	0 (0)	0 (0)	0 (0)		
Normal weight: [18.5;25]	65 (27.3)	2 (10.0)	67 (26.0)		
Overweight: [25;30]	92 (38.7)	12 (60.0)	104 (40.3)		
Moderate obesity: [30;35]	61 (25.6)	5 (25.0)	66 (25.6)		
Severe obesity: [35;40]	15 (6.3)	0 (0)	15 (5.8)		
Morbid or massive obesity: ≥40	5 (2.1)	1 (5.0)	6 (2.3)	*0.1561*	NA
WHO score at day 1, *n* (%)					
WHO 2/3	170 (71.4)	15 (71.4)	185 (71.4)		
WHO 4	68 (28.6)	6 (28.6)	74 (28.6)	*1*	1.000 [0.372; 2.685]
Comorbidities, *n* (%)					
No	111 (46.6)	4 (19)	115 (44.4)		
Yes	127 (53.4)	17 (81)	144 (55.6)	*0.0147*	3.715 [1.214; 11.368]

**Figure 2:**
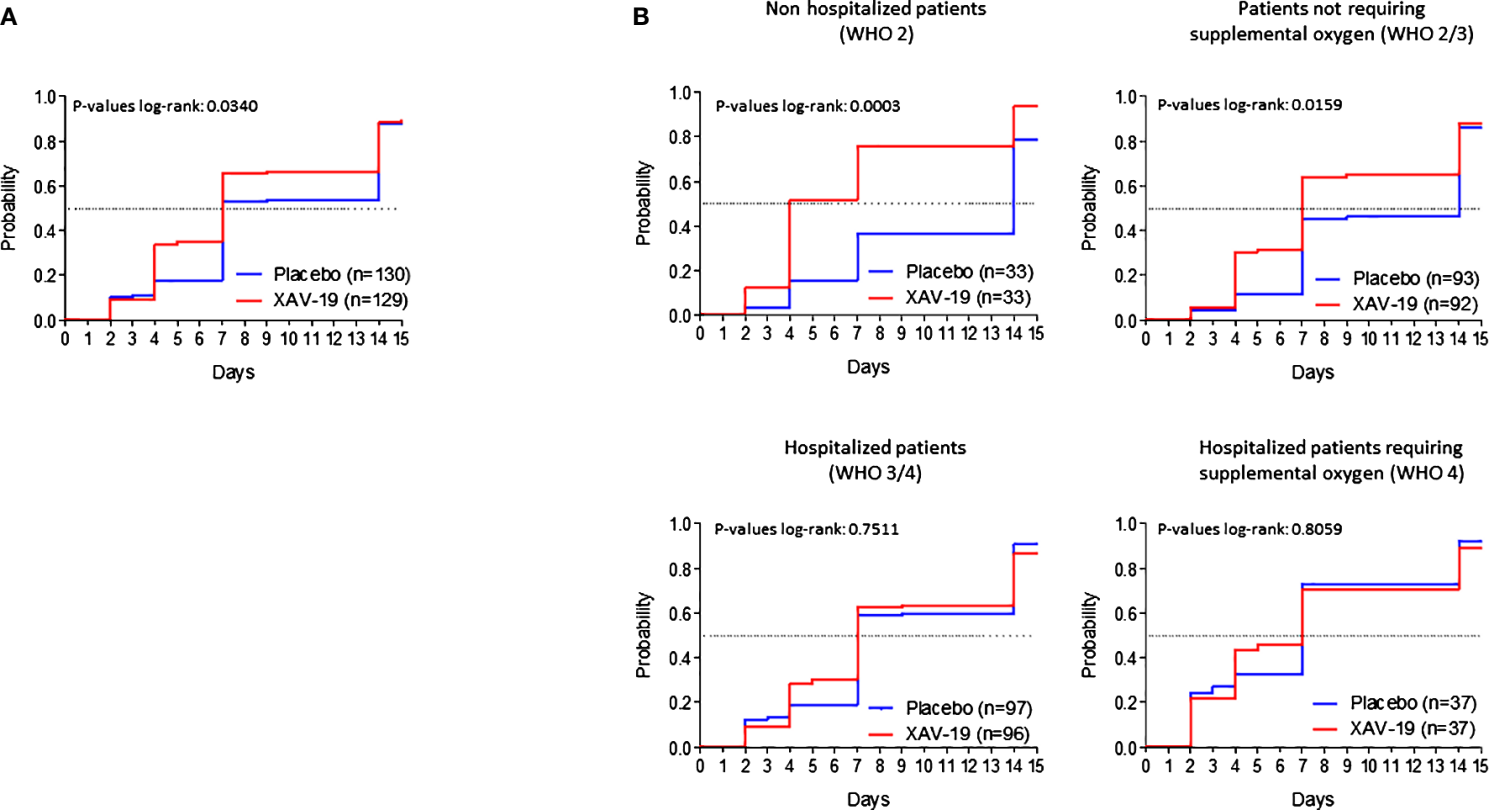
Time to clinical improvement. Kaplan–Meier curves by randomization group on the whole TARGET population **(A)** or on the TARGET population with a score of 2, 2 to 3, 3 to 4, or 4 at baseline **(B)**. Improvement was defined as a diminution of at least one point on the WHO score compared to the WHO score at day 1. Dotted lines represent the median time to improvement.

Secondary endpoints: the proportion of patients with an aggravation of two points at day 8 was 5.4% and 6.2% in the XAV-19 and placebo groups, respectively. No difference was observed according to the time of symptom onset. At day 15, aggravation of at least one point/two points was similar at 4%/3.2% and 2.4%/2.4%, respectively. Time to aggravation did not differ between the two groups, and no difference was detected according to the WHO score at randomization (not shown). The proportion of patients with COVID improvement at day 15 was similar between the XAV-19 and placebo arms (41.4% vs. 46.3%, respectively, *p* = 0.623) (not shown). However, improvement did occur earlier in the XAV-19 arm (*p* = 0.0340) (**Figure 2A**). This benefit was more significant for non-hospitalized patients (WHO score of 2, median time to improvement at 4 days vs. 14, *p* = 0.0003) or for any patients not requiring supplemental oxygen at day 1 (WHO score of 2 or 3, median time at 7 days vs. 14, *p* = 0.0159) while not significant for hospitalized patients (WHO score of 3 or 4) or hospitalized patients requiring supplemental oxygen (WHO score of 4) (*p* = 0.7511 and *p* = 0.8059) (**Figure 2B**). Length of hospital stay, number of patients referred to ICU, and number of patients with mechanical ventilation were not significantly different between the two groups (not shown). Overall survival at day 15 was 99.2% in the XAV-19 group, and 96.9% in the placebo group (*p* = 0.370, not significant). Until day 29, 5 deaths were reported in each group.

### Safety

A total of 83 AEs (45 in the XAV-19 arm and 38 in the placebo arm) were observed from 29 patients in each group (**Table 3**). The percentage of mild/moderate AE in the XAV-19 arm was 91% (vs. 76.3% in the placebo arm). A total of 22 SAEs were reported in the XAV-19 group compared to 13 in the placebo group. None of them were considered to be related by the investigators. Interestingly, SAEs >3 account for 86% of SAEs in the XAV-19 arm vs. 92% in the placebo arm. The number of fatal SAEs was equivalent between the two groups; five patients died in each group, from respiratory failure, multi-organ failure, or disease progression. No anaphylaxis or hypersensitivity reactions and no infusion-related events were reported in any patients receiving XAV-19.

**Table 3:** Summary of adverse events and serious adverse events in the safety population of the EUROXAV trial.

	XAV-19 (*N* = 139)		Placebo (*N* = 140)	
	Number of patients	Number (%) of AEs/SAEs	Number of patients	Number (%) of AEs/SAEs
**Any adverse event, *N* (%)**	29	45 (54.2)	29	38 (45.8)
Mild/moderate AE, *N* (%/number of AE)		41 (91.1)		29 (76.3)
Related to XAV-19 or placebo, *N* (%/number of AE)	8	9 (20.0)	5	5 (13.2)
**Any SAE, *N* (%)**	10	22 (62.9)	9	13 (37.2)
SAE>3 N (%/number of SAE)	9	19 (86.3)	8	12 (92.3)
SAE related	0	0	0	0
**Infusion-related events**	0	0	0	0
**Death**	5		5	
Cause of death				
Respiratory failure	3		4	
Disease progression	0		1	
Multiorgan failure	1		0	
Cytokine storm	1		0	

### XAV-19 activity on SARS-CoV-2 variants

**Figure 3:**
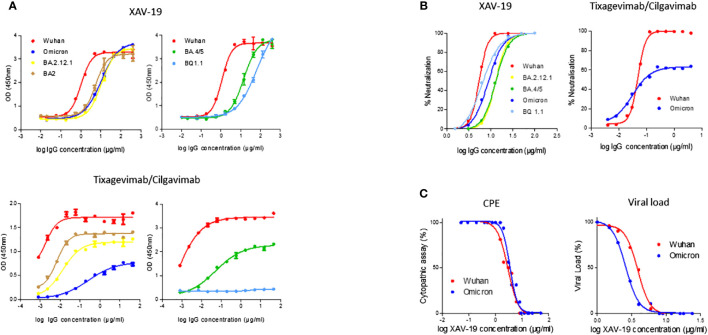
XAV-19 antiviral activity against Omicron and its subvariants. **(A)** Binding of XAV-19 or Evusheld to Wuhan or Omicron and its subvariant RBD: XAV-19 or Evusheld was added to RBD-coated plates at the indicated concentration and revealed with an HRP-conjugated secondary anti-pig or anti-human antibody. **(B)** Neutralizing activity of XAV-19 or Evusheld to Wuhan or Omicron and its subvariant RBD: recombinant His-Tag-RBD pre-incubated with XAV-19 or Evusheld was added to human ACE2-coated plates. Bound RBD was then detected with HRP-conjugated anti-His-Tag antibody. **(C)** Neutralizing activity of XAV-19 on whole replicating viruses: Vero E6 cells were infected with Wuhan or Omicron SARS-CoV-2 strains. CPE was assessed by microscopy examination and viral load percentage was determined by quantitative RT-PCR.

**Figure 4:**
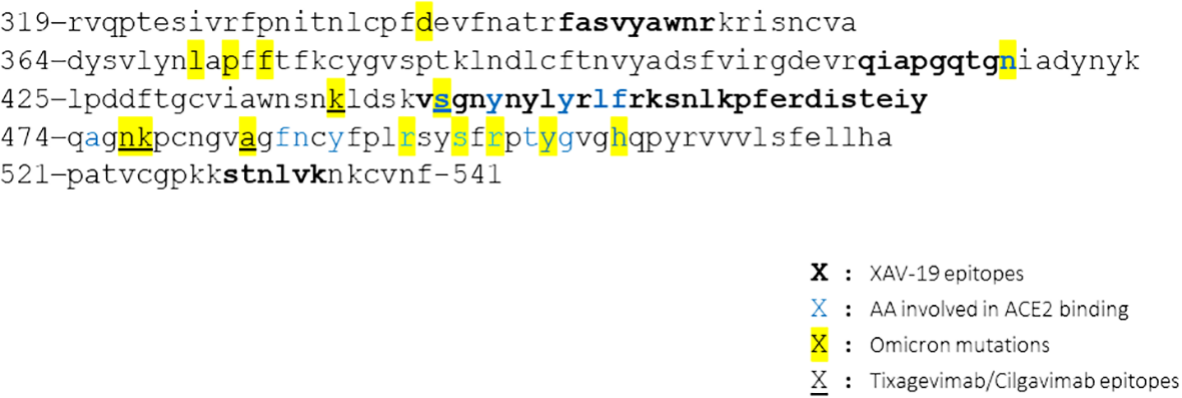
XAV-19 target epitopes lie outside the Omicron mutation sites. Amino acid sequence of the SARS-CoV-2 spike RBD variant Omicron and XAV-19 target epitopes (amino acid sequence numbered according to DBSOURCE sequence reference NC_045512.2). Bold: XAV-19 target epitopes confirmed by proteolytic epitope mapping; blue: amino acids in contact with ACE-2; yellow: mutations found in Omicron, differentiating from the original Wuhan RBD; underlined: tixagevimab/cilgavimab target epitopes.

Binding of XAV-19 to Omicron, BA.2, BA.2.12.1, BA.4/5, and BQ.1.1 RBD appeared slightly lower than binding to the original strain RBD. Yet, a similar plateau was reached regardless of the variant tested (**Figure 3A**, top). In comparison, binding of the association tixagevimab/cilgavimab (Evusheld^®^) to Omicron or BA.4/5 RBD was dramatically reduced, and binding to BQ.1.1 RBD was completely abolished. Binding of BA.2 and BA.2.12.1 subvariants was less affected, with a lower plateau (**Figure 3A**, bottom). Neutralization by XAV-19 was then tested in an RBD/ACE-2 binding competition assay. Comparable dose response profiles with full neutralization by XAV-19 were obtained with all the variants tested (**Figure 3B**, left); IC_50_ was moderately increased (5.9 ± 0.2 and 9.1 ± 0.6 µg/mL for Wuhan and Omicron RBD, respectively). The neutralization efficacy of tixagevimab/cilgavimab was limited to 60% for Omicron versus 100% for Wuhan RBD (**Figure 3B**, right). Neutralization of viral infectivity by XAV-19 was confirmed by CPE assay using clinical isolates. XAV-19 neutralized 100% of CPE for all the variants tested and reached 100% viral load reduction (**Figure 3C**). Interestingly, NT50 against Omicron was in the low range in CPE and lower than for all other variants. Four main target epitopes of XAV-19 (347-fasvyawnr-417, 409-qiapgqtgn-417, 445-vsgnynylyrlfrksnlkpferdisteiy-473, and 530-stnlvk-535) were identified on Omicron RBD by proteolytic epitope mapping (**Figure 4**, bold), including 6 amino acids out of the 17 (in blue) directly involved in ACE-2 receptor binding. Among the 15 Omicron mutations in RBD (in yellow), 2 were shown to lie inside the major XAV-19 target epitopes (N417 and S446) compared to 5 for the association tixagevimab/cilgavimab (underlined; K440, S446, N477, K478, and A484) (22).

## Discussion

Although vaccination is now the primary option against SARS-CoV-2, therapeutic drugs are still needed for immunocompromised or at-risk individuals. Transplant recipients or patients with cancer present indeed an increased susceptibility to infections associated with a higher risk for progression to severe COVID-19 and a lower response to vaccination (23–25). In these patients, prophylactic treatment with mAb such as the tixagevimab/cilgavimab combination reduced the rate of infection and disease severity (26–28). Yet, the potential of anti-SARS-COV-2 mAb is today largely dampened by the continual emergence of resistant variants (8, 9, 29), and as SARS-CoV-2 will probably continue to mutate, robust and variant-resistant treatments are still necessary. XAV-19 is known to neutralize the original Wuhan and the Alpha, Beta, Gamma, and Delta variants (15). Here, we confirmed its neutralizing activity against Omicron and its subvariants, while the association tixagevimab/cilgavimab (Evusheld^®^) lost its activity, as previously published (29). Interestingly, XAV-19 even neutralized the BQ.1.1 subvariant, against which most mAbs or cocktails of mAb are devoid of activity (29, 30). Owing to its high number of mutations (30 mutations in the spike protein, 15 in the RBD, but less than 3% of XAV-19 target epitopes), Omicron showed resistance to more than 80% of the therapeutic antibody candidate (31). The transmissibility of SARS-CoV-2 results from a fine balance between affinity to ACE-2 and the capacity to escape immune response. The need to maintain sufficient affinity to ACE-2 probably limits SARS-CoV-2 RBD mutability as shown by the R493Q reversion mutation observed on the Omicron variants (32). Whereas antibody avidity allows SARS-CoV-2 variability to be overcome (31), our data confirm that targeting multiple alternative epitopes permits pAbs like XAV-19 to keep their binding and neutralization capacity (15).

The therapeutic potential of XAV-19 was investigated in the phase II/III multicenter, randomized, double-blind EUROXAV study. A total of 279 patients were randomized, with an initial WHO score of 2 (outpatients), 3 (hospitalized without oxygen), or 4 (hospitalized under low-flow oxygen). Treatment with XAV-19 was safe and well tolerated. No patient who received XAV-19 had anaphylaxis or hypersensitivity reactions, confirming the interest of our glyco-humanization strategy. Nevertheless, cytokine release or antidrug antibody production cannot be excluded, and a thorough immunogenicity monitoring will have to be performed in the next trials to further validate XAV-19 safety. The number of AEs was similar between groups, and although the number of SAEs was higher in the XAV-19 group, none were found related to the treatment. Our trial suffered from a low inclusion rate and was stopped early, on DSMB advice. The lower proportion of worsening cases, despite the fact that most patients were not vaccinated, might be due to the improvement in the management of patients with COVID-19 (33, 34) and/or to the Omicron strain, known to be less pathogenic than the original one (35). This dramatically lowered the power of the study; thus, difference in disease aggravation (primary endpoint) was detectable between the two arms, whatever the stage. This observation could also be due to the late administration of this antibody, the median of symptom onset being at 5 days, while antibodies are expected to be efficient before day 5 of the infection (36). Our findings seem to confirm the absence of antibodies’ benefit for inpatients. Nevertheless, we showed that XAV-19 efficiently modulated the kinetics of COVID-19 disease as it accelerated the clinical improvement of patients not requiring oxygen therapy (WHO score ≤4). In that respect, our data match recent studies showing the suitability of therapeutics antibodies in patients with mild to moderate COVID-19. Altogether, our data support XAV-19 as a possible and affordable therapeutic option for patients with mild to moderate COVID-19 (36–38). Several challenges are raised by XAV-19 therapy. The main one concerns the selection of appropriate target population. As it is applicable to the early stage of COVID-19, early identification of eligible patients will be crucial. Use of XAV-19 in a prophylactic approach might also be envisaged to overcome this issue. Regarding drug production and in particular control of batch-to-batch reproducibility and viral safety, mastery of production process from animal farming to Ab purification will be mandatory. Today, the absence of a robust, variant-resistant anti-SARS-COV-2 mAb therapy for immunocompromised individuals remains a public health concern (39). High-risk patients might possibly benefit from XAV-19, a hypothesis that needs to be confirmed.

### Limitations

First, the long period of enrollment induces heterogeneity in the management of COVID-19. Second, the choice of the seven-point ordinal scale WHO score as the main endpoint may suffer from discrepancies among practices in the different sites. Standardized criteria for use of oxygen or hospitalization have not been implemented in this trial. Third, although most patients were not able to consent to vaccination, due to the main enrollment period from January to September 2021, this information was not collected during the trial. Next trials should thus consider vaccination status and variant identification. Last, the important reduction of the number of patients has strongly reduced the ability of this trial to detect any efficacy for XAV-19.

### Conclusions

The prematurely ended clinical trial EUROXAV had limited statistical power to analyze the effect of XAV-19, a glyco-humanized anti-SARS-CoV-2 pAb on COVID-19 aggravation for inpatients and outpatients. Nevertheless, XAV-19 significantly improved time to improvement for patients with a WHO score of 2 to 4 and particularly for outpatients. The maintained activity of XAV-19 on variants makes it a potential candidate for passive immunotherapy for immunocompromised patients at the early stage of the disease.

## Data availability statement

The raw data supporting the conclusions of this article will be made available by the authors, without undue reservation.

## Ethics statement

The studies involving humans were approved by The Drug Research Ethics Committee of the Puerta de Hierro Majadahonda Hospital, The Romanian National Bioethics Committee of Medicine and Medical Devices, The Hellenic National Ethics Committee, The Medipol University Clinical Research Ethics Committee and The Bulgarian Ethics Committee for Clinical Trial. The studies were conducted in accordance with the local legislation and institutional requirements. The participants provided their written informed consent to participate in this study.

## Author contributions

GP: Data curation, Investigation, Writing – review & editing. P-JR: Investigation, Writing – original draft, Writing – review & editing. NE: Data curation, Investigation, Writing – review & editing. GE: Investigation, Writing – review & editing. FS: Conceptualization, Data curation, Investigation, Methodology, Supervision, Writing – review & editing. A-GM: Investigation, Writing – review & editing. BV: Conceptualization, Investigation, Methodology, Writing – original draft. OD: Conceptualization, Funding acquisition, Investigation, Methodology, Resources, Supervision, Writing – review & editing. SM: Investigation, Writing – review & editing. VC: Investigation, Writing – review & editing.

## EUROXAV study group

Todor Atanasov, Dan Corneci, Valentin Cuervas-Mons, Gheorghe Lulian Diaconescu, Nikolay Evgeniev, Gerd Fätkenheuer, Elizabeth George, Rosen Georgiev, Benoit Guery, Guillen Santiago Moreno, Bedreag Ovidiu-Horea, Diamantis Kofteridis, Ali Mert, Symeon Metallidis, Hristo Metev, Cristina Mussini, Garyfallia Poulakou, Emmanuel Roilides, Fehmi Tabak, Pilar Vizcarra. DSMB members: Elizabeth George, London, UK; Cristina Mussini, Modena, Italy; Benoît Guéry, Lausanne, Switzerland; Gerd Fätkenheuer, Köln, Germany. Vasiliki Rapti, Panagiotis Chardouvelis, Ioannis Trontzas, Emmanouil Alevrakis, Maria Gangadi, Ourania Koltsida, Georgios Tsoukalas, Ilias Kainis, Vasilios Ntousopoulos, Georgios Kokkotis, Vissaria Sakka.
